# Distribution of *Rachiplusia nu* and *Chrysodeixis includens* in *Bt* and Conventional Soybean Fields in Brazil

**DOI:** 10.3390/insects16040365

**Published:** 2025-04-01

**Authors:** Carolina T. D. Godói, Tamylin K. Ishizuka, Guilherme A. Gotardi, Natália R. F. Batista, Luiz H. Marques, Antônio César S. Santos, Mário H. Dal Pogetto, Timothy Nowatzki, Amit Sethi, Mark L. Dahmer

**Affiliations:** 1 Corteva Agriscience, Rodovia SP 147 Km 71, Mogi Mirim 13801-540, São Paulo, Brazil; carolina.godoi@corteva.com (C.T.D.G.); guilherme.gotardi@corteva.com (G.A.G.); natalia.batista@corteva.com (N.R.F.B.); luiz.marques@corteva.com (L.H.M.); antonio.santos@corteva.com (A.C.S.S.); 2 Corteva Agriscience, 9330 Zionsville, Indianapolis, IN 46268, USA; mario.dal-pogetto@corteva.com; 3 Corteva Agriscience, Johnston, IA 50131, USA; tim.nowatzki@corteva.com (T.N.); amit.sethi@corteva.com (A.S.); mark.dahmer@corteva.com (M.L.D.)

**Keywords:** Plusiinae, Noctuidae, looper, soybean looper, sunflower looper, population dynamics, Cry1Ac, Cry1Ac × Cry1F, SBL, SFL

## Abstract

*Chrysodeixis includens* (Walker, 1858) and *Rachiplusia nu* (Guenée, 1852) are important defoliating pests belonging to the Plusiinae subfamily in the Western hemisphere. *C. includens* is a major lepidopteran pest of soybean in the Americas, whereas, until 2021, *R. nu* was more restricted to the temperate regions of South America. Recently, reports of *R. nu* feeding on Cry1Ac soybean and occurring in tropical regions of Brazil have raised questions regarding the distribution of this species. The morphological similarity of the larvae from the two species makes it difficult to correctly identify the species in the field, which may lead to an underestimation of *R. nu* in Brazilian territory. This study aimed to address these questions by using a molecular approach to identify Plusiinae caterpillars throughout three seasons in non-*Bt*, Cry1Ac, and Cry1Ac × Cry1F soybean fields. Here, we carried out a comprehensive spatial sampling of the primary soybean-producing regions in Brazil. The results showed that *R. nu* has been the main Plusiinae occurring in soybean over the last three years, and it was present in all sampled regions. For Cry1Ac and Cry1Ac × Cry1F soybeans, up to 99% of the samples collected in 2023/24 were identified as *R. nu*. Non-*Bt* soybeans had higher variations in the proportion of the two species among the regions and across seasons, indicating that populations of *C. includens* and *R. nu* are co-occurring throughout the country. This is, to our knowledge, the most robust report assessing the distribution of *C. includens* and *R. nu* in Brazil using a molecular tool. This study provides clarification of *R. nu* occurrence and highlights the importance of pest monitoring from an integrated pest management perspective.

## Simple Summary

The loopers *Chrysodeixis includens* and *Rachiplusia nu* are important agriculture pests in South America. In Brazil, *C. includens* is considered the main defoliating pest of soybean throughout the country, whereas *R. nu* is historically more common in temperate regions. Recently, *R. nu* has been reported in tropical regions, causing injury to *Bt* soybeans, which raised concerns about its distribution range, pest adaptation, and resistance management. Due to the morphological similarity of *C. includens* and *R. nu* in the larval stage, identifying these species in the field is challenging, which may have led to an underestimation of *R. nu*’s presence in Brazil. This study aimed to clarify these issues by sampling caterpillars across the main soybean-producing regions in Brazil over three growing seasons. The results show that *R. nu* is present in all sampled areas of Brazil, and it is now the main pest found on *Bt* soybeans in Brazil, whereas *C. includens*, when present, is found predominantly on non-*Bt* soybean varieties.

## 1. Introduction

The Plusiinae subfamily within the order Lepidoptera comprises many economically important species that impact agriculture [1]. Their larvae, commonly called loopers, are polyphagous and can therefore cause damage to a wide range of hosts, such as soybeans, cotton, sunflowers, and vegetables [2,3,4]. The injury consists mainly of larval feeding on leaf parenchyma, which results in loss of productivity due to the reduced photosynthetic capacity of the attacked plants [5,6].

The most relevant species in South America are the soybean looper, *Chrysodeixis includens* (Walker [1858]) (Lepidoptera: Noctuidae), and the sunflower looper, *Rachiplusia nu* (Guenée, 1852) (Lepidoptera: Noctuidae) [7,8,9]. When in the larval stage, these two species are very similar, and the main morphological difference between them is the presence of two teeth on the inner part of the jaw of *C. includens* and the absence of teeth in *R. nu*, which is visually imperceptible [10]. Reliable species identification can be achieved by distinguishing pupae and adults or using DNA barcoding methods; however, these approaches are challenging in a field environment [10,11].

In Brazil, *Chrysodeixis includens* was considered a secondary pest of soybean until the 1990s [12]. Since then, it emerged as a key pest and spread throughout all soybean-producing regions of the country [13]; this is mainly attributed to the increased use of fungicides to control Asian Soybean Rust and the consequent decrease in natural biological control [14]. Furthermore, this species is more tolerant to insecticides compared to other soybean caterpillars [15,16], and its habit of feeding on the leaves of the lower middle third of the plant makes it difficult to control with insecticides after canopy closure [12,17,18]. Currently, the management of this insect relies mainly on the use of transgenic plants expressing insecticidal proteins [19,20,21].

*Rachiplusia nu*, alternatively, was historically more geographically restricted to temperate regions of South America, playing the role of a key pest of soybean in Uruguay, Paraguay, and Argentina [21,22,23]. Although this species has always occurred as a secondary pest in Brazil [3,8,24], its economic importance was restricted to the southern region, and a large-scale survey showed that, until 2020, its occurrence was low in the main soybean-producing regions of the country [25]. However, from 2021 onward, a loss of sensitivity of the species to the Cry1Ac protein was observed, followed by some reports of outbreaks of moderate density in the central and northeastern regions of Brazil [16,26,27].

There are currently three soybean transgenic events for lepidopteran control commercially available in Brazil. The first *Bt* soybean was launched in 2013, and it contained the events MON 87701 × MON 89788 that express the Cry1Ac protein. However, its durability has been threatened by the presence of field-evolved insect-resistant populations of *Crocidosema aporema* and *Rachiplusia nu* [16,23]. Two new *Bt* soybean products were launched in 2021: the events MON-87751-7 × MON-87701-2 × MON87708 × MON89788, expressing Cry1Ac and Cry1A.105 × Cry2Ab2, and the events DAS-81419-2 × DAS-444Ø6-6, expressing Cry1Ac and Cry1F. *C. includens* is labeled as a target pest for all three technologies, but *R. nu* is not [28,29].

The increasing prevalence of *R. nu* in commercial soybean fields raises concerns about its distribution, adaptability, population dynamics, and response to *Bt* technologies. The difficulty in visually differentiating between *C. includens* and *R. nu* larvae might have led to an underestimation of the occurrence and injury caused by *R. nu* in the country. Therefore, this three-year study aimed to address this issue by verifying, through a molecular identification method, the species composition of *R. nu* and *C. includens* in non-*Bt*, Cry1Ac, and Cry1Ac × Cry1F soybean fields in the main producing regions of Brazil. According to the results, we discuss some practical implications of our findings for the management of these species in Brazil.

## 2. Materials and Methods

### 2.1. Sample Collection

During three consecutive soybean growing seasons (2021/22, 2022/23, and 2023/24), loopers were sampled in 91 geographically distinct soybean fields in Brazil, containing non-*Bt* (Roundup Ready—RR), Cry1Ac (MON 87701 × MON 89788), and Cry1Ac × Cry1F (DAS-81419-2 × DAS-444Ø6-6) technologies. Collections were carried out at random locations based on the presence of looper larvae in soybean fields. There were 23 fields in 2021/22, 30 in 2022/23, and 38 in 2023/24 (Table S1).

**Figure 1:**
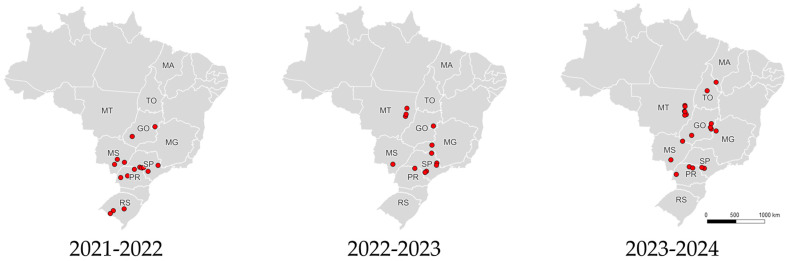
Location of samples collected in the 2021/22, 2022/23, and 2023/24 seasons.

In total, 1601 individuals were collected from 9 regions across Brazil: Goiás/DF (GO/DF), Maranhão (MA), Mato Grosso (MT), Mato Grosso do Sul (MS), Minas Gerais (MG), Paraná (PR), Rio Grande do Sul (RS), São Paulo (SP), and Tocantins (TO) (Figure 1, Table 1). The samples collected in the Federal District (DF) were grouped with those from the Goiás state.

The loopers were collected using beat sheets during the vegetative and reproductive stages of soybean (V2-R6). The individuals sampled from each field were stored in 50 mL labeled conical centrifuge tubes containing 70% alcohol. The tubes were stored in thermal packaging, sent to the laboratory, and kept at −20 °C until DNA extraction. The number of samples collected in each technology varied according to the occurrence of caterpillars in the field, and only samples with at least 4 individuals were considered.

All insect collections were made under the System of Authorization and Information on Biodiversity (SISBIO) license number 58435 and 89321.

### 2.2. Species Identification

Total genomic DNA was extracted from segments of larval body using the DNeasy Plant Mini Kit (Qiagen, Hilden, Germany), following the manufacturer’s instruction. The species identification of field-collected individuals was determined through the end-point PCR amplification of a mitochondrial cytochrome c oxidase subunit I (mt-COI) gene fragment. A set of species-specific primers was employed: a common reverse primer, MMRC_1955 (5′-CAGATCTACCACCATGAGCAATA-3′), and species-specific forward primers, MMRC_1964 (5′-GGATTTGGTAATTGACTTGTACCTCTT-3′) for *C. includens* and MMRC_1988 (5′-TCCTGGATCTTTAATTGGAGAT-3′) for *R. nu* [30]. The resulting amplicons of distinct sizes—199 bp for *C. includens* and 299 for *R. nu*—were visualized on agarose gel. Reactions were carried out in 25 μL total volume, using 30 ng of genomic DNA, 0.5 μM of each primer, and 12.5 μL of GoTaq G2 2X Master Mix (Promega, Madison, WI, USA). The PCR conditions included an initial incubation at 95 °C (2 min), followed by 35 cycles of 95 °C (30 s), 56 °C (20 s), 72 °C (30 s), and a final incubation of 72 °C for 2 min. After amplification, 5.0 μL was loaded in 2% agarose gel stained with SYBR Safe DNA Gel Stain (Invitrogen, Carlsbad, CA, USA).

### 2.3. Statistical Analysis

All the statistical analyses were performed in R software—R Version 4.4.0 [31]. The *t*-test function was used to perform a two-sided paired *t*-test (α = 0.05) for comparisons between C. includens and R. nu larval counts for each soybean type and season.

A descriptive analysis of the proportion of species collected in each region, soybean type, and season was performed. The number of individuals per species sampled in each state was represented as a percentage of the total.

## 3. Results

**Figure 2:**
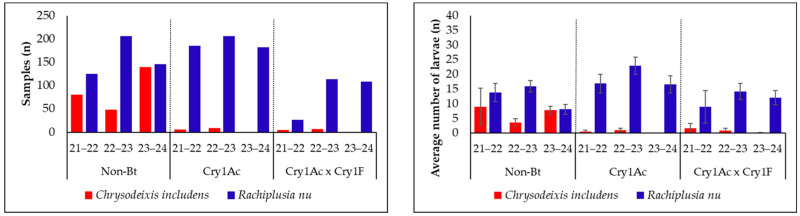
Proportion (**left**) and number (**right**) of *Rachiplusia nu* and *Chrysodeixis includens* individuals sampled during the 2021/22, 2022/23, and 2023/24 seasons in Non-*Bt*, Cry1Ac, and Cry1Ac × Cry1F soybean fields in Brazil. Vertical bars represent standard errors of the mean values.

A total of 430 loopers were collected and identified using DNA analysis in the 2021/22 season, 592 in the 2022/23 season, and 579 in the 2023/24 season. Of the 1601 larvae sampled, 81.4% were identified as *R. nu*. The comparison between the proportion and number of samples identified by each season and technology is shown in Figure 2 and Table 2. The sampling number in each soybean technology reflected the abundance of larvae in natural infestations. A higher number of non-*Bt* soybean fields were sampled, followed by Cry1Ac, and then Cry1Ac × Cry1F. An average of 17.6 ± 1.0 loopers were sampled per field.

The 2021/22 season exhibited the highest prevalence of *C. includens,* accounting for 21.4% of the collected samples, compared to 10.8% in 2022/23 and 21.1% in 2023/24. The average number of larvae in each technology and season is shown in Table 2. According to these results, during the 2021/22 and 2023/24 seasons, there was no significant difference between the average number of individuals of *C. includens* and *R. nu* in non-*Bt* fields (*p* = 0.585). *R. nu* was the main species found in Cry1Ac × Cry1F technology in the 2022/23 (*p* = 0.004) and 2023/24 (*p* = 0.001) seasons, while, in the 2021/22 season, there was no significant difference between species occurrence (*p* = 0.390). Collections made in Cry1Ac soybean resulted in most of the samples identified as *R. nu* in all three seasons.

**Figure 3:**
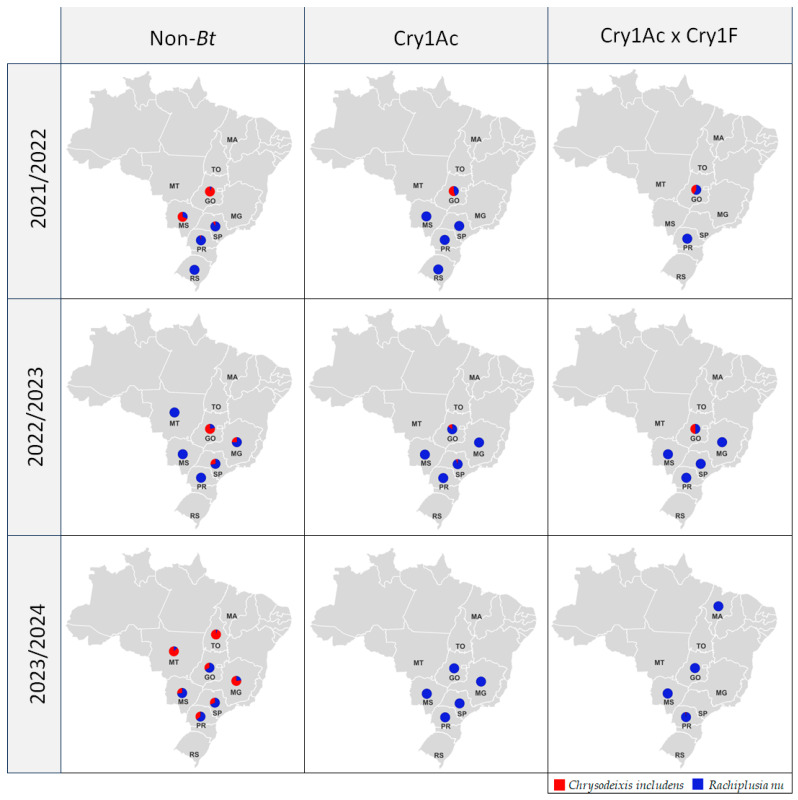
Locations and proportion of samples identified as *Chrysodeixis includens* and *Rachiplusia nu* in Non-*Bt*, Cry1Ac, and Cry1Ac × Cry1F soybean.

Species composition varied in non-*Bt* soybean across the years, in contrast to soybeans expressing *Bt* proteins, which were colonized primarily by *R. nu* (Figure 3). Although *R. nu* was, in general, the prevalent species across all seasons, *C. includens* was the main species in non-*Bt* soybean in the samples from Goiás and Mato Grosso do Sul in 2021/22, from Goiás in 2022/23, and from Mato Grosso, Tocantins, and Minas Gerais in 2023/24 season.

The data from non-*Bt* soybean provided a clearer understanding of the distribution of both species in the sampled regions. The first season of the survey indicated that *Rachiplusia nu* was predominant over *C. includens* in the southern states of Brazil, whereas *C. includens* was still the main species in the Center West. In the second year, *R. nu* occurrence expanded and increased considerably in the Center West, while *C. includens* started to increase in the Southeast. Finally, in the third year of the survey, the species composition acquired a more mixed pattern, with the occurrence of *C. includens* increasing in all the sampled states of Brazil.

Samples from Cry1Ac fields also showed a predominance of *R. nu* over *C. includens*, equivalent to 96.9% of samples in the 2021/22 season, 95.8% in 2022/23, and 100% of samples in the 2023/24. In the 2021/22 season, *C. includens* was the main species in the state of Goiás (54.5%), while, in the other states, 100% of specimens were identified as *R. nu*. In 2022/23, *C. includens* was present in a small portion of the samples collected in Goiás and São Paulo.

For the Cry1Ac × Cry1F technology, *C. includens* represented 41.7% of the samples from Goiás in 2021/22 and 50% in 2022/23. In the 2023/24 season, one individual out of 110 sampled was *C. includens*, in Paraná State, which represents 0.9% of all samples for this technology. Over the three-year study period, a decline in the occurrence of *C. includens* was noted, with a decrease from 15.6% in 2021/22, to 5.8% in 2022/23, and further to 0.9% in 2023/24. Among the 293 samples collected in *Bt* technologies during 2023/24, 292 consisted of *R. nu*.

## 4. Discussion

This survey offers the most extensive sampling with reliable identification of loopers in Brazil reported to date. We sampled 1601 individuals in a total of nine states of Brazil over three seasons (2021–2024) in the main soybean-producing regions of Brazil. *R. nu* specimens were detected in all the collections.

Compared to *C. includens* occurrence in non-*Bt* soybean, a significantly higher number of *R. nu* larvae was observed in the 2022/23 season but not in the 2021/22 and 2023/24 seasons. Therefore, the occurrence of *R. nu* in Brazil was comparable to or higher than *C. includens* in the last three years. Furthermore, data collected from non-*Bt* soybean offered a better understanding of the expansion of *R. nu* in each region and over the years. In 2021/22, a low occurrence of *R. nu* was detected in Goiás and Mato Grosso do Sul. However, a change in this scenario was observed in 2022/23, with an increase in *R. nu* occurrence in the Cerrado region. Then, in 2023/24, *C. includens* was identified as the most common species in non-*Bt* soybean in Mato Grosso, Tocantins, and Minas Gerais but not in Goiás. Moreover, the occurrence of *C. includens* increased also in the southern states of Brazil, indicating that the species composition acquired a more mixed pattern. Altogether, these results are evidence of the rapid adaptation and spread of *R. nu* to tropical regions of Brazil, eventually surpassing *C. includens* in some locations.

As for the samplings in *Bt* soybean fields, we observed a predominance of *R. nu* over *C. includens*, reaching close to 100% of the samples in 2023/24. The Goiás region registered a divergent result in the 2021/22 and 2022/23 seasons, where the majority of Cry1Ac and half of the Cry1Ac × Cry1F samples were identified as *C. includens*. These data were attributed to the high infestation of *C. includens* in the region, as observed in the samples from non-*Bt* fields. Because of the higher abundance of *C. includens* in Goiás samples, in addition to the low occurrence of larvae in Cry1Ac × Cry1F soybean, there was no significant difference between the number of *C. includens* and *R. nu* larvae collected in Cry1Ac × Cry1F in 2021/22 season. Nevertheless, several studies have demonstrated that these proteins provide efficient control of *C. includens* in Brazil [23,29,32,33,34].

Our results provide complementary, novel information to previous studies comparing the species composition of Plusiinae in Brazil. Collections made from 2016 to 2018 indicated that *R. nu* was not detected in samples from Paraná and Mato Grosso do Sul, and until then, this species was still restricted to its endemic region of occurrence [12]. In 2019 and 2020, this species comprised only 0.6% of the Lepidopteran pests in non-*Bt* soybean [25]. Then, in the 2020–2021 season, *R. nu* was present in all states of the Mid-South of Brazil, although less abundant than *C. includens* [23]. Our data from 2021 to 2024 indicated that *R. nu* not only had successfully been established in the Center West of Brazil, up to the northeast in Maranhão state, but it had done so in comparable numbers to *C. includens.* These results consolidate *R. nu* as a key pest of soybean in Brazil.

We suggest three factors that likely contributed to the successful establishment, increase, and dispersal of *R. nu* populations towards the Brazilian Cerrado: first, the selection pressure for resistant populations imposed by the high adoption of *Bt* soybean [23,26]; second, the year-round availability of hosts, including cover crops during the off-season, followed by intense soybean planting in the summer [35]; and third, the reduced use of insecticides that could have previously been aiding the suppression of lepidopteran pest populations in the field [36].

Monitoring and diagnosing target species in the field are important tools for the decision-making and successful implementation of Integrated Pest Management (IPM) strategies [37]. Our study showed that both *C. includens* and *R. nu* co-occurred in non-*Bt* soybean fields, while *R. nu* was the prevalent species in *Bt* soybean. These findings have important implications for pest management. *C. includens* and *R. nu* exhibit differential susceptibility to chemical and biological insecticides [16], and while field-evolved resistance to insecticides has been reported in *C. includens* [15,38,39], *R. nu* populations showed low levels of resistance to major insecticides in Brazil [40]. Additionally, commercial biopesticide formulations containing baculovirus or *Bacillus thuringiensis* isolates are available in Brazil for *R. nu* and *C. includens* control [41]. As we move towards a more sustainable agriculture system, more alternatives for managing these species will be offered in the biological control space. Finally, cultural practices have shown to play a key role in altering pest status; therefore, changes in the cropping system on a broader scale can also contribute to IPM programs in Brazil.

Taking all this into account, efforts should be directed to the continuous monitoring and characterization of field populations. Monitoring allowed the early detection of the reduced susceptibility of *R. nu* to Cry1Ac in 2017 [42], and since then, outbreaks of resistant populations have been reported in Brazil [23,26,43], Argentina [44], and Uruguay [45]. Although the genetic basis and the adaptive costs associated with resistance to Cry1Ac have been characterized [44,46,47,48], additional studies are still needed to better understand the resistance of *R. nu* to Cry1F. As for *C. includens*, this study confirmed that *Bt* soybean has been efficient in controlling natural populations; however, this pest is still widespread throughout Brazil. Therefore, monitoring *C. includens* susceptibility to Cry1Ac and Cry1Ac × Cry1F soybean remains essential in terms of resistance management.

## Figures and Tables

**Table 1:** Number of larvae collected in soybean fields in each region of Brazil.

Region	2021/22			2022/23			2023/24		
	Non-*Bt*	Cry1Ac	Cry1Ac × Cry1F	Non-*Bt*	Cry1Ac	Cry1Ac × Cry1F	Non-*Bt*	Cry1Ac	Cry1Ac × Cry1F
Goiás/DF	65	11	12	14	20	14	80	45	23
Maranhão	0	0	0	0	0	0	0	0	7
Mato Grosso	0	0	0	65	0	0	42	0	0
Mato Grosso do Sul	19	60	0	20	20	20	41	44	36
Minas Gerais	0	0	0	20	40	20	30	4	0
Paraná	30	20	20	20	20	20	56	69	44
Rio Grande do Sul	13	22	0	0	0	0	0	0	0
São Paulo	79	79	0	116	116	47	16	21	0
Tocantins	0	0	0	0	0	0	21	0	0
Total	206	192	32	255	216	121	286	183	110

**Table 2:** Number of individuals and proportion of samples identified as *Chrysodeixis includens* and *Rachiplusia nu* in Non-*Bt*, Cry1Ac, and Cry1Ac × Cry1F soybean. n = number of total larvae from all locations. N = number of fields sampled. Mean = average number of samples collected per field. Means with * indicate a statistical difference in the occurrence of *R. nu* and *C. includens* in each soybean type (*p* < 0.05).

Season	Soybean Technology	n	N	Mean ± SE	*Chrysodeixis includens* (%)	*Rachiplusia nu* (%)
2021/22	Non-*Bt*	206	9	22.9 ± 4.8	39.3	60.7
	Cry1Ac	192	11	17.5 ± 3.0 *	3.1	96.9
	Cry1Ac × Cry1F	32	3	10.7 ± 4.7	15.6	84.4
	Total	430	23	18.7 ± 2.5	21.4	78.6
2022/23	Non-*Bt*	255	13	19.6 ± 1.2 *	18.8	81.2
	Cry1Ac	216	9	24.0 ± 2.7 *	4.2	95.8
	Cry1Ac × Cry1F	121	8	15.1 ± 2.6 *	5.8	94.2
	Total	592	30	19.7 ± 1.3	10.8	89.2
2023/24	Non-*Bt*	286	18	15.9 ± 2.0	49.0	51.0
	Cry1Ac	183	11	16.6 ± 2.9 *	0.0	100.0
	Cry1Ac × Cry1F	110	9	12.2 ± 2.4 *	0.9	99.1
	Total	579	38	15.2 ± 1.4	21.1	78.9
Total (2021–2024)		1601	91	17.6 ± 1.0	24.4	75.6

## Data Availability

The original contributions presented in this study are included in the article/Supplementary Material. Further inquiries can be directed to the corresponding author.

## Supplementary Materials

The following supporting information can be downloaded at: https://www.mdpi.com/article/10.3390/insects16040365/s1; Table S1: Sampling information. Figure S1: Proportion by state of *R. nu* and *C. includens* samples found in Non-Bt, Cry1Ac, and Cry1Ac × Cry1F soybean in the seasons of 2021–2022 (A), 2022–2023 (B) and 2023–2024 (C).
