# Supplementary material for: Distribution of Rachiplusia nu and Chrysodeixis includens in Bt and Conventional Soybean Fields in Brazil
Source: Insects. 2025 Apr 1;16(4):365. doi: 10.3390/insects16040365 (PMC12027875; doi:10.3390/insects16040365)
Supplement: Supplementary file 1 [file insects-16-00365-s001.zip › insects-3520806-supplementary.pdf]

Table S1. Sampling information.

Technology	City, State	Latitude	Longitude
**Season 2021-22**			
Non-*Bt*	Planaltina, Distrito Federal	15° 43' 31.336" S	47° 36' 27.846" W
	Rio Verde, Goiás	17° 25' 15.960" S	51° 34' 49.080" W
	Dourados, Mato Grosso do Sul	22° 17' 00.240" S	54° 39' 20.160" W
	Cambé, Paraná	23° 08' 05.280" S	51° 11' 57.840" W
	Santana do Livramento, Rio Grande do Sul	30° 50' 15.360" S	55° 23' 43.080" W
	Mogi Mirim, São Paulo	22° 26' 59.280" S	47° 04' 24.600" W
	Ourinhos, São Paulo	22° 53' 57.120" S	49° 49' 57.000" W
	Parapanema, São Paulo	23° 29' 50.280" S	48° 48' 57.240" W
	Salto Grande, São Paulo	22° 52' 18.480" S	49° 57' 08.640" W
Cry1Ac	Planaltina, Distrito Federal	15° 43' 31.336" S	47° 36' 27.846" W
	Rio Verde, Goiás	17° 25' 15.960" S	51° 34' 49.080" W
	Anaurilândia, Mato Grosso do Sul	21° 55' 32.880" S	52° 55' 54.689" W
	Nova Alvorada do Sul, Mato Grosso do Sul	21° 24' 32.501" S	54° 10' 23.221" W
	Assis Chateaubriant, Paraná	24° 34' 29.280" S	53° 35' 02.760" W
	Cachoeira do Sul, Rio Grande do Sul	30° 01' 25.760" S	52° 59' 59.880" W
	Rosário Do Sul, Rio Grande do Sul	30° 20' 04.840" S	54° 52' 11.640" W
	Mogi Mirim, São Paulo	22° 26' 59.220" S	47° 04' 24.450" W
	Palmital, São Paulo	22° 46' 12.731" S	50° 16' 08.432" W
	Santa Cruz do Rio Pardo, São Paulo	22° 49' 15.348" S	49° 35' 07.440" W
	Santa Cruz do Rio Pardo, São Paulo	22° 49' 58.808" S	49° 35' 17.880" W
Cry1Ac x Cry1F	Planaltina, Distrito Federal	15° 43' 31.336" S	47° 36' 27.846" W
	Rio Verde, Goiás	17° 25' 15.960" S	51° 34' 49.080" W
	Campo Mourão, Paraná	24° 16' 37.092" S	52° 23' 25.512" W
**Season 2022-23**			
Non-*Bt*	Planaltina, Distrito Federal	15° 38' 43.800" S	47° 36' 38.520" W
	Indianópolis, Minas Gerais	18° 57' 31.680" S	47° 51' 23.040" W
	Dourados, Mato Grosso do Sul	22° 16' 59.117" S	54° 39' 19.174" W
	Água Boa, Mato Grosso	13° 57' 22.320" S	52° 27' 22.680" W
	Canarana, Mato Grosso	13° 35' 51.360" S	52° 21' 08.280" W
	Querencia, Mato Grosso	12° 33' 41.760" S	52° 12' 44.280" W
	Sertaneja, Paraná	22° 59' 45.319" S	50° 50' 14.010" W
	Aguaí, São Paulo	22° 04' 57.720" S	47° 0' 52.200" W
	Guará, São Paulo	20° 21' 35.920" S	47° 55' 00.120" W
	Itaberá, São Paulo	23° 42' 14.483" S	49°0 3' 27.619" W
	Mogi Mirim, São Paulo	22° 26' 59.220" S	47° 04' 24.450" W
	Parapanema, São Paulo	23° 30' 20.570" S	48° 48' 42.347" W
	Salto Grande, São Paulo	22° 52' 17.760" S	49° 57' 08.604" W
Cry1Ac	Planaltina, Distrito Federal	15° 38' 43.800" S	47° 36' 38.520" W
	Indianópolis, Minas Gerais	18° 57' 31.680" S	47° 51' 23.040" W
	Dourados, Mato Grosso do Sul	22° 16' 59.117" S	54° 39' 19.174" W
	Sertaneja, Paraná	22° 59' 45.319" S	50° 50' 14.010" W
	Aguaí, São Paulo	22° 04' 57.720" S	47°0 0' 52.200" W
	Guará, São Paulo	20° 21' 35.920" S	47° 55' 00.120" W
	Itaberá, São Paulo	23° 42' 14.483" S	49°0 3' 27.619" W
	Parapanema, São Paulo	23° 30' 20.570" S	48° 48' 42.347" W
	Salto Grande, São Paulo	22° 52' 17.760" S	49° 57' 08.604" W
Cry1Ac x Cry1F	Planaltina, Distrito Federal	15° 38' 43.700" S	47° 36' 38.600" W
	Indianópolis, Minas Gerais	18° 57' 31.560" S	47° 51' 23.100" W

	Dourados, Mato Grosso do Sul	22° 16' 59.117'' S	54° 39' 19.174'' W
	Sertaneja, Paraná	22° 59' 45.319'' S	50° 50' 14.010'' W
	Aguaí, São Paulo	22° 04' 57.890'' S	47° 00' 52.320'' W
	Guará, São Paulo	20° 21' 35.000'' S	47° 55' 00.000'' W
	Itaberá, São Paulo	23° 42' 14.483'' S	49° 03' 27.619'' W
	Salto Grande, São Paulo	22° 52' 17.760'' S	49° 57' 08.604'' W
**Season 2023-2024**			
Non-*Bt*	Planaltina, Distrito Federal	15° 43' 14.160'' S	47° 36' 29.160' 'W
	Rio Verde, Goiás	17° 45' 33.840'' S	51° 01' 59.160'' W
	Cristalina, Goiás	16° 38' 56.760'' S	47° 37' 04.440'' W
	Luziânia, Goiás	16° 21' 23.040'' S	47° 45' 14.040'' W
	Água Boa, Mato Grosso	14° 08' 07.080'' S	51° 58' 16.680'' W
	Querencia, Mato Grosso	12° 33' 49.200'' S	52° 29' 52.100'' W
	Querencia, Mato Grosso	12° 44' 04.920'' S	52° 11' 05.280'' W
	Canarana, Mato Grosso	13° 34' 22.200'' S	52° 16' 19.300'' W
	Canarana, Mato Grosso	13° 37' 27.800'' S	52° 12' 32.000'' W
	Água Boa, Mato Grosso	14° 15' 01.400'' S	52° 13' 43.400'' W
	Dourados, Mato Grosso do Sul	22° 16' 58.980'' S	54° 39' 21.730'' W
	Chapadão do Sul, Mato Grosso do Sul	18° 46' 18.430'' S	52° 37' 12.750'' W
	Paracatu, Minas Gerais	16° 59' 35.300'' S	46° 46' 00.400'' W
	Maripá, Paraná	24° 32' 46.000'' S	53° 42' 28.000'' W
	Rolândia , Paraná	23° 13' 25.522'' S	51° 27' 09.522'' W
	Assaí, Paraná	23° 26' 09.420'' S	50° 50' 46.241'' W
	Parapanema , São Paulo	23° 30' 06.451'' S	48° 48' 50.648'' W
	Palmas, Tocantins	10° 24' 10.404'' S	48° 21' 38.077'' W
Cry1Ac	Planaltina, Distrito Federal	15° 43' 14.160'' S	47° 36' 29.160'' W
	Rio Verde, Goiás	17° 45' 33.840'' S	51° 01' 59.160'' W
	Cristalina, Goiás	16° 38' 56.760'' S	47° 37' 04.440'' W
	Luziânia, Goiás	16° 21' 23.040'' S	47° 45' 14.040'' W
	Dourados, Mato Grosso do Sul	22° 16' 58.980'' S	54° 39' 21.730'' W
	Chapadão do Sul, Mato Grosso do Sul	18° 46' 18.430'' S	52° 37' 12.750'' W
	Paracatu, Minas Gerais	16° 59' 35.300'' S	46° 46' 00.400'' W
	Maripá, Paraná	24° 32' 46.000'' S	53° 42' 28.000'' W
	Rolândia , Paraná	23° 13' 25.522'' S	51° 27' 09.522'' W
	Assaí, Paraná	23° 26' 09.420'' S	50° 50' 46.241'' W
	Tejupá, São Paulo	23° 20' 58.159'' S	49° 15' 42.792'' W
Cry1Ac x Cry1F	Rio Verde, Goiás	17° 45' 33.840'' S	51° 01' 59.160' 'W
	Cristalina, Goiás	16° 38' 56.760'' S	47° 37' 04.440'' W
	Luziânia, Goiás	16° 21' 23.040'' S	47° 45' 14.040'' W
	Balsas, Maranhão	08° 30' 39.532'' S	46° 46' 02.050'' W
	Dourados, Mato Grosso do Sul	22° 16' 58.980'' S	54° 39' 21.730'' W
	Chapadão do Sul, Mato Grosso do Sul	18° 46' 18.430'' S	52° 37' 12.750'' W
	Maripá, Paraná	24° 32' 46.000'' S	53° 42' 28.000'' W
	Rolândia, Paraná	23° 13' 25.522'' S	51° 27' 09.522'' W
	Assaí, Paraná	23° 26' 09.420'' S	50° 50' 46.241'' W

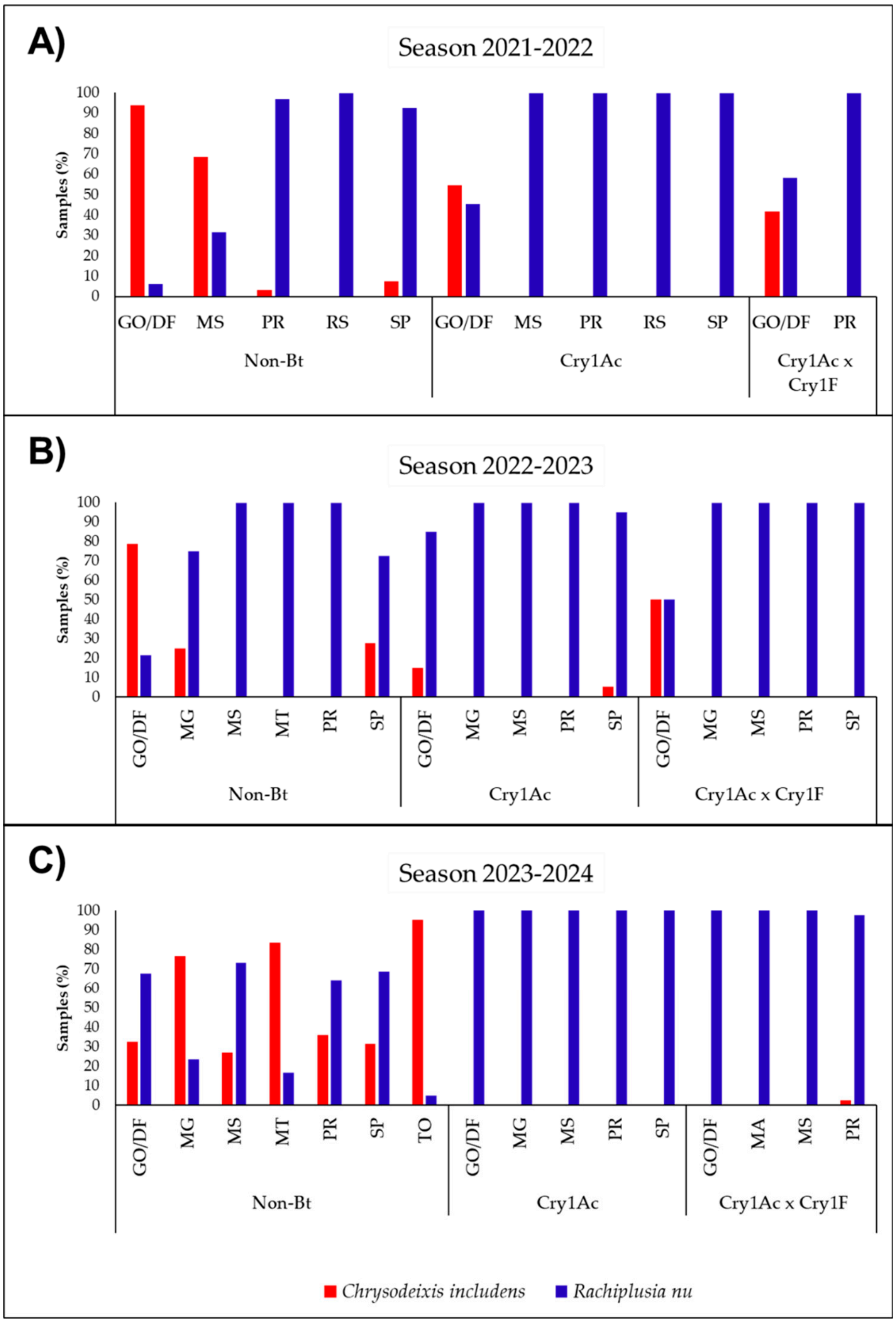

Figure S1. Proportion by state of *R. nu* and *C. includens* samples found in Non-*Bt*, Cry1Ac, and Cry1Ac x Cry1F soybean in the seasons of 2021–2022 (**A**), 2022–2023 (**B**) and 2023–2024 (**C**).
